# Disentangling Gut Bacterial Community Patterns in *Cryptocercus punctulatus* and Comparing Their Metagenomes with Other Xylophagous Dyctioptera Insects

**DOI:** 10.3390/insects16111128

**Published:** 2025-11-04

**Authors:** Mercedes Berlanga, David Miñana-Galbis, Ricardo Guerrero

**Affiliations:** 1 Departament de Biologia, Sanitat i Medi Ambient, Secció de Microbiologia, Facultat de Farmàcia i Ciències de l’Alimentació, Universitat de Barcelona, 08028 Barcelona, Spain; davidminyana@ub.edu; 2 Laboratori de Microbiologia Molecular i Antimicrobians, Departament de Patologia i Experimental Terapèutica, Facultat de Medicina, Universitat de Barcelona, 08907 Hospitalet del Llobregat, Spain

**Keywords:** *Cryptocercus*, xylophagous dyctioptera insects, gut microbiota, functional microbiome

## Abstract

Gut microbiota enable wood-feeding insects to digest recalcitrant diets. Two DNA-based analyses were performed. Amplicon sequencing of gut microbiota samples from *Cryptocercus punctulatus* showed inter-individual heterogeneity with visually distinct ordination patterns; however, no statistically significant differences were detected. Shotgun metagenomics was used to compare the taxonomic and functional profiles of *C. punctulatus* gut microbiota with those of other xylophagous Dictyoptera. Despite taxonomic differences, *C. punctulatus* microbiota revealed functional convergence with termites (*Mastotermes darwiniensis* and *Nasutitermes* sp.). Carbohydrate metabolism was performed by different bacterial phyla across all insects. All insect species possessed metabolic potential for cellulose, hemicellulose, pectin, and starch digestion, but lignin degradation capabilities were not detected. Termites showed higher abundance of chitin and xylan degradation pathways and nitrogen fixation genes, though nitrogen fixation was also present in *Cryptocercus* cockroaches. Genes for oxidative stress tolerance were present across all species but were most abundant in cockroaches, particularly, *Cryptocercus*. All insects harbored antibiotic resistance genes, with highest levels found in cockroaches. These findings indicate that metabolic requirements for wood digestion shape gut microbial community assembly across xylophagous insects, with distinct microbial taxa contributing to cellulose and hemicellulose breakdown. Moreover, the widespread presence of antibiotic resistance genes raises concerns about the potential transmission of antibiotic resistance within insect-associated microbiomes.

## Simple Summary

Xylophagous insects, such as cockroaches and their close relatives, feed on wood, a food source that is very difficult to digest because it is primarily composed of lignin and cellulose, contains plant defense chemicals, and provides very little nitrogen, an essential nutrient. To survive on such a poor diet, these insects rely on the help of their gut microbiota, which supply enzymes to break down plant fibers, detoxification and antioxidant functions to cope with harmful chemicals, and even include bacteria capable of adding nitrogen to their food supply. In our study, we compared the gut bacteria of different wood-feeding insects and found that, while each species possessed unique microbial communities, they shared similar functional traits essential for breaking down wood and conserving nitrogen. We also observed that all insect gut microbiota contained antibiotic resistance genes, with cockroaches—including *C. punctulatus*—harboring the highest levels. These findings enhance our understanding of how insects thrive on such extreme diets and underscore the role of gut microbes in recycling plant material in nature. This knowledge may also inform new strategies for converting plant waste and addressing the global challenge of antibiotic resistance.

## 1. Introduction

Xylophagous insects are specialized on lignocellulose-rich wood diets that present important challenges: lignin, crystalline cellulose, diverse phenolics and secondary metabolites (e.g., alkaloids, coumarins, flavonoids, stilbenes, tannins, terpenoids, tropolones), and extremely low nitrogen content. To overcome these difficulties, their gut microbiota includes specialized bacterial taxa such as *Clostridia*, *Bacteroidia*, and Gammaproteobacteria that produce a suite of cellulolytic and hemicellulolytic enzymes (e.g., endoglucanases, xylanases) critical for breaking down cellulose and hemicellulose. Additionally, genera like *Pseudomonas* and *Burkholderiales* contribute to oxidative modification of lignin and possess antioxidant enzymes that help detoxify harmful plant secondary metabolites [1,2,3,4]. Given the complexity of lignocellulose digestion and the specialized adaptations required, understanding the gut microbiota of specific xylophagous lineages provides crucial insights into evolutionary digestive strategies.

The chemical composition of wood and herbaceous plants is variable, but generally contains 50–65% carbohydrates such as hemicellulose and cellulose, 20–25% phenolic compounds (e.g., lignin, monolignols, and other phenolic compounds), and 20% other components such as secondary metabolites. Regarding nitrogen content, woody plant tissues usually contain only 0.03–0.10% nitrogen [5,6]. The C/N ratio of most woody species is between 350 and 500:1 and can be as high as 1250:1 in some conifer species [6]. Cellulose and hemicellulose polymers require specialized enzymes for their digestion [7]. Relevant enzymes (provided by the host and the symbiotic microbiota) involved in hydrolysis include endoglucanases, beta-glucosidases, xylanases, hemicellulases, esterases and other glycoside hydrolases. The lignin contains functional groups such as carbonyl, methoxyl, phenolic hydroxyl, and alcoholic hydroxyl groups that greatly impact its reactivity [6]. Degradation of lignin can result in the production of oxygen radicals that may increase oxidative stress, creating a hostile environment for microorganisms [7].

These challenging nutritional constraints have driven the evolution of specialized digestive strategies across different insect lineages, most notably within the superorder Dictyoptera, a well-established insect lineage that includes termites, cockroaches, and mantids. Six termite families and the wood-feeding cockroach genus *Cryptocercus* share the ability to digest lignocellulose with a complex consortium of gut bacteria and protists, whereas the higher-termite family Termitidae has lost its protists and relies solely on bacteria [8]. Despite their shared evolutionary history and lignocellulose diets, comprehensive comparative analyses of gut microbiota across xylophagous Dictyoptera remain scarce. Most research on wood-feeding Dictyoptera, especially termites, uses certain cockroach lineages as evolutionary reference points rather than conducting broad, systematic comparative analyses across all wood-feeding groups. *Cryptocercus* cockroaches are the primary model for termite evolution due to their close phylogenetic relationship and shared traits, while other wood-feeding Dictyoptera are less frequently included in comparative frameworks [9,10]. While termites mainly consume wood, many cockroaches exhibit diverse diets, including omnivorous scavenging, feeding on leaf litter, and decaying wood [11,12]. Notable lignocellulose-eating cockroaches are *Cryptocercus* spp. from East Asia and North America, *Parasphaeria boleiriana* from Brazil [13], and *Panchlora nivea* from the Caribbean. *Cryptocercus* and *Parasphaeria* inhabit rotting logs, while *Panchlora* lives in decomposing palm trunks, leaf litter, and refuse piles of fungus-farming ants [14]. This ecological diversity suggests corresponding variation in gut microbiome composition and function.

The basic digestive tract architecture (foregut, midgut, hindgut) is conserved across insects, yet its physicochemical heterogeneity (pH, redox potential, oxygen gradients) structures distinct microbial communities [4,15]. In cockroaches, the hindgut is typically anoxic, neutral to slightly alkaline and mildly reducing [11]. The microbiome substantially influences digestion and nutrient absorption: germ-free *Periplaneta americana* individuals exhibit underdeveloped digestive organs, which normalize after re-inoculation with conspecific gut microbes [16].

The aims of this study were to elucidate the bacterial gut normobiota of the wood-feeding cockroach *C. punctulatus* and to compare its microbiota composition and predicted functional capabilities with those of other xylophagous Dictyoptera insects, particularly termites (*Mastotermes darwiniensis*—“lower” termite and *Nasutitermes* sp.—“higher” termite). The significance of this work lies in advancing the understanding of evolutionary digestive strategies in insects that feed on lignocellulose-rich wood diets, which are difficult to digest due to the presence of complex polymers like lignin and cellulose, plant defense chemicals, and very low nitrogen content. The gut microbiota is crucial for these insects to digest wood by providing specialized enzymes for fiber breakdown, detoxification, nitrogen fixation, and antioxidant functions. Despite their close phylogenetic relatedness, comprehensive comparative analyses across xylophagous Dictyoptera—including cockroaches and termites—are scarce. Comparative analyses of termite and cockroach microbiomes could help the understanding of lignocellulose adaptation across Dictyoptera, and have applied implications for sustainable agriculture and biotechnology related to lignocellulose bioconversion.

## 2. Materials and Methods

### 2.1. Sample Collection and DNA Extraction

*Cryptocercus punctulatus* specimens were collected in Virginia, USA, by Dr. Michael Dolan (University of Massachusetts, Amherst, MA, USA) and maintained under laboratory conditions for several months, using the same type of wood from which the cockroaches had been collected in the forest. Three adult *C. punctulatus* cockroaches were transported to our laboratory in Barcelona, Catalonia, Spain, for analysis. During transport, the insects were kept in tubes at room temperature. Upon arrival, the specimens exhibited vitality, activity, and an apparent health status adequate for experimental procedures. The cockroaches were immediately dissected under sterile conditions to extract the intact gut. Briefly, the abdomens were opened using a sterile scalpel, and the entire gut was collected and placed into an Eppendorf tube for subsequent DNA extraction. Disinfection and dissection were performed in a laminar flow cabinet to avoid contamination.

The gut tissue was homogenized using a FastPrep system with 0.1 mm glass beads. Bulk DNA was then extracted by repeated phenol–chloroform washing [13]. Following homogenization, an equal volume of phenol–chloroform–isoamyl alcohol solution (typically 25:24:1, *v*/*v*/*v*, Thermo Fisher Scientific, Madrid, Spain) was added to the homogenate to denature proteins and separate nucleic acids. The mixture was shaken and centrifuged at 12,000× *g* for 10 min at room temperature to separate phases. The upper aqueous phase containing DNA was carefully transferred to a new tube. This phenol–chloroform extraction step was repeated 2–3 times until the interphase was clear, indicating thorough removal of proteins and lipids. After the final extraction, DNA was precipitated by adding 0.1 volume of 3 M sodium acetate (pH 5.2) (Thermo Fisher Scientific) and 2 volumes of cold 100% ethanol (Thermo Fisher Scientific), followed by incubation at –20 °C for at least 1 h or overnight. The sample was then centrifuged at 12,000× *g* for 10 min at 4 °C to pellet the DNA. The pellet was washed with 70% ethanol to remove residual salts, air-dried briefly, and resuspended in an appropriate volume of TE buffer (Thermo Fisher Scientific).

### 2.2. Cryptocercus punctulatus Metagenome Sequencing and Data Processing

Samples were pooled, and shotgun metagenomic sequencing was performed using the Illumina MiSeq_RunMicro300 cycles (2 × 150) platform by the Genomic and Bioinformatic Service of the Autonomous University of Barcelona (sample Cp_shotgun).

Paired-end fastq file sequences generated by Illumina MiSeq were assembled using the MEGAHIT tool in Galaxy Version 1.2.9 [https://usegalaxy.eu/] [17]. The assembled sequences were then submitted to the DOE-JGI Metagenome Annotation Pipeline (MAP v.4) [https://gold.jgi.doe.gov/] [18]. For genomic assembly, the Velvet algorithm package was used. The pipeline runs against curated models, derived from full-length genes within IMG, while keeping the best-scoring models. The identification of protein-coding genes was performed using a consensus of four different ab initio gene prediction tools: prokaryotic GeneMark.hmm (v.2.8), MetaGeneAnnotator (v. Aug 2008), Prodigal (v. 2.6.2) and FragGeneScan. Protein-coding genes with translations shorter than 32 amino acids were deleted. Assignment was made at 90% of the KO gene sequence covered by the alignment [18]. KO genes refer to genes that have been assigned a KEGG Orthology (KO) identifier within the KEGG (Kyoto Encyclopedia of Genes and Genomes) database. The use of MAP for metagenomic analysis enabled the detection of partial genes associated with various functional activities. However, given these limitations, the functional annotations should be considered preliminary and interpreted as putative activity potential.

Data available in the JGI database Gold pj. Ga0134290 (*C. punctulatus*) (https://gold.jgi.doe.gov/analysis_project?id=Ga0134290 (accessed on 1 November 2025). General statistics from JGI: Reads were assembled into 177,102 scaffolds, reflecting a highly fragmented metagenome. Quality filtering by JGI indicated that over 80% of bases achieved Q30 or higher, suggesting reasonable base accuracy. Among all predicted coding sequences, 18.5% matched KEGG Orthology (KO) terms, indicating moderate annotation coverage common to fragmented gut metagenomes. Ribosomal RNA genes were rare (0.05% of 16S and 0.18% of 18S rRNA). BLAST (version 2.13.0)-based taxonomic classification at 60% identity cutoff yielded 20% bacterial hits, 2% eukaryotic, and roughly 78% unassigned, the latter representing short or chimeric contigs and poorly conserved sequences. The high proportion of unclassified scaffolds and low rRNA recovery indicate incomplete coverage of microbial diversity. These limitations restrict the precision of gene and pathway abundance estimates but still provide valuable community-level insights into the *C. punctulatus* gut microbiome.

### 2.3. Downloaded Data for Comparative Analysis

Several *C. punctulatus* 16S rRNA amplicon datasets were retrieved from the NCBI Bioproject Database. Sample Cp (Bioproject PRJDB7102) [19]; Cp_1, Cp_2 and Cp_3 (Bioproject PRJNA238270) [20]; Sample Cp_4 (Bioproject PRJNA217467) [21], and Sample Cp_5 (Bioproject PRJNA284583) [13] (Table 1).

Several gut microbiome shotgun metagenomes of different xylophagous Dictyoptera species were retrieved from the JGI-GOLD database. Their associated information is summarized in Table 2.

### 2.4. Bioinformatics and Statistical Analyses

Amplicon reads were processed by the amplicon analysis pipeline of the SILVA base data (http://www.arb-silva.de). The pipeline automatically performs stringent quality filtering steps: All reads are aligned to the SILVA reference database using the SINA aligner. Reads that fail to align properly (non-rDNA fragments, chimeras, poor similarity, or mismatched orientation) are discarded. Reads that pass alignment undergo checks for minimum length, ambiguous bases (N > 2%) and homopolymers (>8 bp). Taxonomic abundance tables were generated at multiple ranks, with a focus on the phylum level. Relative abundances were calculated by expressing the read counts assigned to each phylum as a percentage of the total classified reads per sample. Differences in microbiota composition between groups were assessed using the Kolmogorov–Smirnov test for non-parametric data. Statistical significance was determined at *p* < 0.05. Alpha diversity was measured by the Shannon index. Shannon’s index *H* is an estimator of taxa diversity (combining richness and evenness) and was computed in R using the vegan package [22]. For amplicon sequencing, principal coordinate analysis (PCoA) plots of normalized sample community distributions were generated using Bray–Curtis dissimilarity and analyzed within the MTP platform of EzBio-Cloud Apps (ChunLab Inc., Seoul, Republic of Korea) (https://www.EZbiocloud.net/).

For each insect host species, metagenomic datasets obtained from the JGI GOLD database and the present study were processed using the JGI Metagenome Annotation Pipeline, which includes sequence quality control, gene prediction, taxonomic classification, and functional annotation against the Kyoto Encyclopedia of Genes and Genomes (KEGG) Orthology (KO) database. Data analyzed by the JGI metagenome annotation pipeline resulted in very low resolution for eukaryotic organisms.

For shotgun metagenome samples, Bray–Curtis dissimilarity was used to measure beta diversity and produce PCoA plots via the web-based JGI Genomes Online Database (https://gold.jgi.doe.gov/).

Functional potential was assessed by compiling KO annotations from the JGI genomes online database. KO abundance values were normalized to relative abundance (% of total annotated genes) to account for differences in sequencing depth.

## 3. Results

### 3.1. Bacterial Gut Patterns in Cryptocercus punctulatus

**Figure 1:**
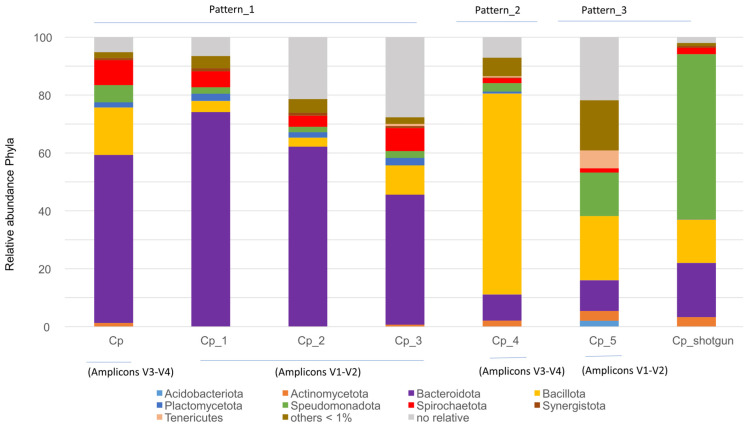
Relative abundance of the major bacterial phyla in the gut microbiota of *C. punctulatus* across seven samples. Samples include Cp, Cp_1–Cp_5, and Cp_shotgun sample (this study). Three bacterial community patterns were observed, with no significant differences among groups (Kolmogorov–Smirnov test, *p* > 0.05).

The gut bacterial microbiota of multiple *C. punctulatus* samples collected from natural environments in Virginia and North Carolina, USA (Cp, Cp_1, Cp_2, Cp_3, Cp_4 and Cp_5) were compared with that of *C. punctulatus*, Cp_shotgun (this study). No significant differences were detected by the Kolmogorov–Smirnov test; however, three distinct gut bacterial patterns were identified: Pattern-1 in Cp, Cp_1, Cp_2, and Cp_3; Pattern-2 in Cp_4; and Pattern-3 in Cp_5 and Cp_shotgun. Pattern-1 was characterized by a high proportion of Bacteroidota; Pattern-2, by Bacillota and Pattern-3, by an increased proportion of Pseudomonadota (Figure 1, Supplementary Figure S1).

Microbial diversity across the identified gut patterns, measured using the Shannon index, was 3.10 for Pattern-1 (Cp, Cp_1, Cp_2 and Cp_3), 4.515 for Pattern-2 (Cp_4), and 3.826 for Pattern-3 (Cp_5). Diversity analysis is commonly used to assess gut microbiota health. However, establishing a universally accepted definition remains challenging due to individual variation, diet, lifestyle, host genetics, and environmental factors [23]. In this study, the absence of significant differences among the patterns indicates that maintaining the samples Cp_5 and Cp_shotgun under laboratory conditions did not substantially alter their gut microbiota composition.

After establishing these bacterial gut patterns, we compared shotgun-derived taxa among different xylophagous insects.

### 3.2. Shotgun Taxa of Cryptocercus punctulatus and Other Xylophagous Dyctioptera

**Figure 2:**
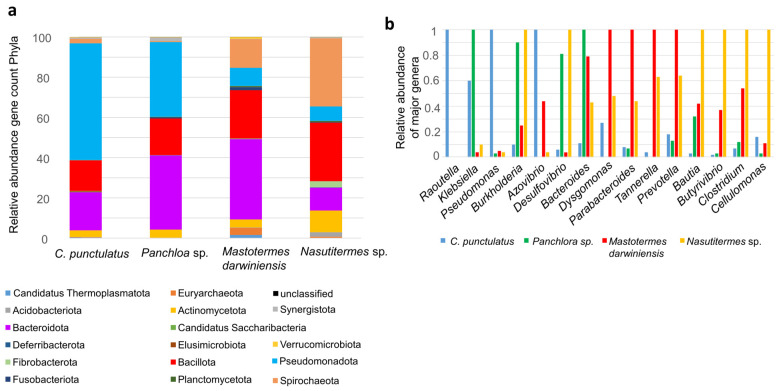
Shotgun taxa of *C. punctulatus* and other xylophagous Dyctioptera. (**a**) The bar chart showed the relative abundance of bacterial phyla present in the gut microbiota of four insect species: *C. punctulatus*, *Panchlora* sp., *M. darwiniensis*, and *Nasutitermes* sp. The relative abundance is presented as gene count percentages for each phylum, as indicated by the color key below the chart. (**b**) Relative abundance of major genera identified in the four insect gut metagenomes. For each genus, the maximum number of detected sequences among all species was assigned a relative value of 1. Abundances in the other species are shown relative to this maximum value, facilitating cross-species comparisons of dominant genera.

Shotgun metagenomics exhibited limited resolution for detecting protist-related sequences, most of which are unique to certain termite families and the related cockroach genus *Cryptocercus* [15]. Therefore, our analysis focused on bacterial microbiota diversity and functionality. Shotgun metagenomic profiling revealed that the most abundant bacterial phyla in *C. punctulatus* were Pseudomonadota (57.4%), Bacteroidota (18.8%), Bacillota (15%), Actinomycetota (3.2%) and Spirochaeota (2.2%). Archaea accounted for less than 1% of relative abundance, belonging primarily to Methanobacteriota (e.g., “*Candidatus* Methanoplasma”, *Methanobacterium* and *Methanobrevibacter*) (Figure 2a). Among Pseudomonadota Gamma- and Betaproteobacteria predominated. The dominant Gammaproteobacteria families were *Enterobacteriaceae* (e.g., *Raoultella* and *Klebsiella*) and *Pseudomonadaceae* (e.g., *Pseudomonas*). *Rhodocyclaceae* represented 63% of Betaproteobacteria. The four most abundant Bacteroidota families were *Bacteroidaceae* (23.3%), *Tannerellaceae* (23.4%), *Dysgonomonadaceae* (17.7%) and *Prevotellaceae* (9.6%). Within Bacillota, the *Clostridia* class constituted 64.4% primarily *Lachnospiraceae* and *Clostridiaceae*.

Shotgun metagenomes from other Dictyoptera species exhibited distinct compositions. In the cockroach *Panchlora* sp., the predominant phyla were Pseudomonadota (36.7%), Bacteroidota (36.5%) and Bacillota (18.1%). The lower termite *Mastotermes darwiniensis* was dominated by Bacteroidota (38.9%), Bacillota (23.5%), Spirochaeota (13.8%) and Pseudomonadota (8.8%). The higher termite *Nasutitermes* sp. exhibited greater abundances of Spirochaeota (33.2%), Bacillota (28.6%), Bacteroidota (11.2%), Actinomycetota (10.6%) and Pseudomonadota (7%) (Figure 2a).

At >60% gene identity, genus-level distributions differed markedly between *C. punctulatus* and other Dictyoptera. In *C. punctulatus*, the most abundant genera were *Raoultella*, *Klebsiella*, *Pseudomonas*, *Azovibrio* and *Dysgomonas*. In *Panchlora* sp. Dominant genera included *Klebisella*, *Burkholderia*, *Desulfovibrio* and *Bacteroides*. The lower termite *M. darwiniensis* was enriched in *Dysgomonas*, *Parabacteroides*, *Tannerella* and *Prevotella*, whereas the higher termite *Nasutitermes* sp. was dominated by *Burkholderia*, *Desulfovibrio*, *Blautia*, *Butyrivibrio*, *Clostridium* and *Cellulomonas*. *Spirochaete* genera such as *Treponema* were also more prevalent in *Nasutitermes* sp. and *M. darwiniensis* (Figure 2b).

Principal coordinate analysis (PCoA) based on family-level taxa showed that *C. punctulatus* harbors a distinct microbiota compared with both termites and the more distantly related cockroach *Panchlora* sp. (Supplementary Figure S2a). However, PCoA clustering based on KEGG orthologs (KO) profiles showed that *C. punctulatus* grouped closely with termites, particularly the phylogenetically ancient lower termite *M. darwiniensis* (Supplementary Figure S2b).

### 3.3. Functional Nutritional Shotgun Metagenome of Cryptocercus punctulatus and Other Xylophagous Dyctioptera

**Figure 3:**
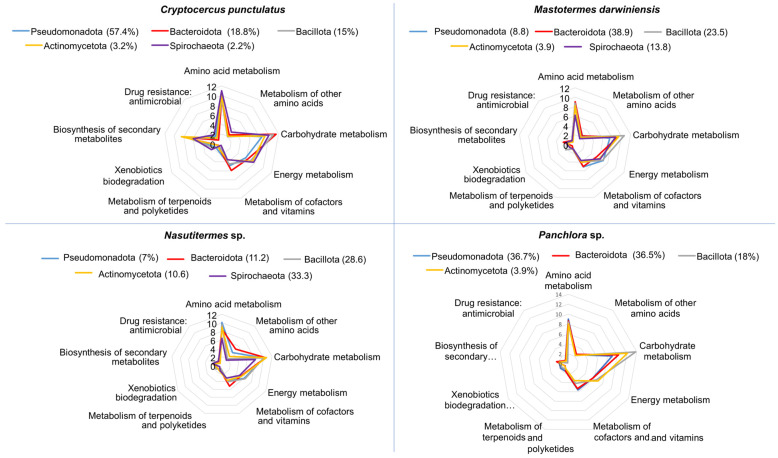
Radial plots depicting the predicted functional potential of the major bacterial phyla within the gut microbiota of four insect species: *C. punctulatus*, *M. darwiniensis*, *Nasutitermes* sp., and *Panchlora* sp. For each host, the four most abundant phyla are displayed, with their relative abundance indicated in parentheses. Each axis in the radial graphs represents a major metabolic or functional category predicted for the bacterial community, including: amino acid metabolism, metabolism of other amino acids, carbohydrate metabolism, energy metabolism, metabolism of cofactors and vitamins, metabolism of terpenoids and polyketides, xenobiotic biodegradation, biosynthesis of secondary metabolites, and antimicrobial drug resistance. Values indicate the frequency of gene functions predicted for each pathway, based on metagenomic data.

Shotgun metagenomics was used to evaluate the functional potential of the gut microbiota in *C. punctulatus*. Predicted proteins were classified into KEGG orthologs (KOs) and mapped to the dominant bacterial phyla. Functional analysis revealed that the major gut bacteria in the host primarily participate in amino acid metabolism, carbohydrate metabolism, energy production, and the metabolism of vitamins and cofactors. To a lesser extent, they contribute to terpenoid and polyketide metabolism and to xenobiotic degradation (Figure 3). Although the overall metabolic profiles were broadly similar across Dictyoptera examined, the specific bacterial taxa performing these functions differed. For example, carbohydrate metabolism in *C. punctulatus* was primarily associated with Bacteroidota, whereas in *M. darwiniensis* and *Panchlora* sp. it was mainly linked to Bacillota, and in *Nasutitermes* sp. to Actinomycetota and Bacteroidota (Figure 3). Genes related to xenobiotic detoxification represented a minor fraction of the relative total KO abundance. These findings suggest that core gut bacterial roles involve nutrient provisioning, lignocellulose digestion, and, to a lesser degree, detoxification.

**Figure 4:**
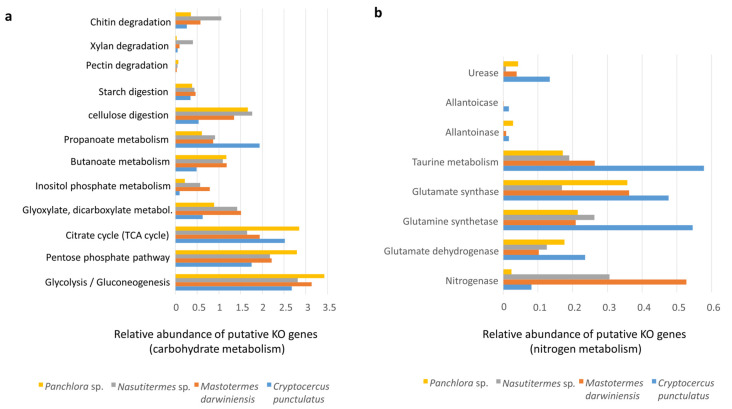
Putative KO genes related to carbohydrate metabolism and nitrogen metabolism. (**a**) Bar graph showing the relative abundance of putative KO genes related to diverse carbohydrate metabolic pathways analyzed by shotgun metagenomics in the gut microbiota of *Panchlora* sp. (yellow), *Nasutitermes* sp. (grey), *Mastotermes darwiniensis* (orange), and *C. punctulatus* (blue). Metabolic pathways evaluated include chitin, xylan, pectin, starch, and cellulose degradation, etc. (**b**) Bar graph displaying the relative abundance of putative KO genes involved in nitrogen metabolism for the same four insect species.

Individual KO genes detected were grouped into functional categories (e.g., carbohydrate and nitrogen pathways). Because each category comprises a different number of putative KOs, comparisons are valid only within categories. Putative genes for carbohydrate hydrolysis were identified across all insect microbiomes examined, supporting the digestion of cellulose, starch, pectin, xylan, chitin, and butanoate (Figure 4a). The inferred capacities for chitin and xylan degradation were higher in *Nasutitermes* sp. than in the cockroaches and *M. darwiniensis* (Figure 4a). Inositol phosphate metabolism was less represented in cockroaches than in termites, with similar trends observed for glyoxylate and dicarboxylate metabolism (Figure 4a). Short-chain fatty acids such as propionate and butyrate produced from dietary carbohydrates by gut microbes may be utilized by the host. Notably, propionate metabolism was particularly well represented in *C. punctulatus*.

Nitrogen acquisition strategies, including nitrogen fixation and nitrogenous waste recycling, were also examined (Figure 4a). Putative nitrogen fixation via the nitrogenase complex was higher in termites; however, in *C. punctulatus*, it may also represent an important nitrogen source (Figure 4b), partly due to nitrogen-fixing ectosymbionts (*Treponema* spirochaetes and members of the Bacteroidales) associated with *Barbulanympha* and *Trichonympha* protists, as well as other diazotrophic gut microorganisms [15,24]. Genes encoding urease, allantoicase, and allantoainase were detected in *C. punctulatus* and *Panchlora* sp. and *M. darwiniensis*. *C. punctulatus* exhibited higher urease activity, likely reflecting the role of the endosymbiont *Blattabacterium*. In this study, *Blattabacterium* accounted for approximately 3.3% of the total community, a subset of the 18% assigned to Bacteroidota, possibly reflecting contamination from the fat body, which houses *Blattabacterium* during intestinal dissection. Because the fat body closely envelops the intestine in cockroaches, inadvertent disruption during dissection can release *Blattabacterium* into gut samples [25] (Figure 4b).

### 3.4. Functional Stress and Antibiotic Shotgun Metagenome of Cryptocercus punctulatus and Other Xylophagous Dyctioptera

**Figure 5:**
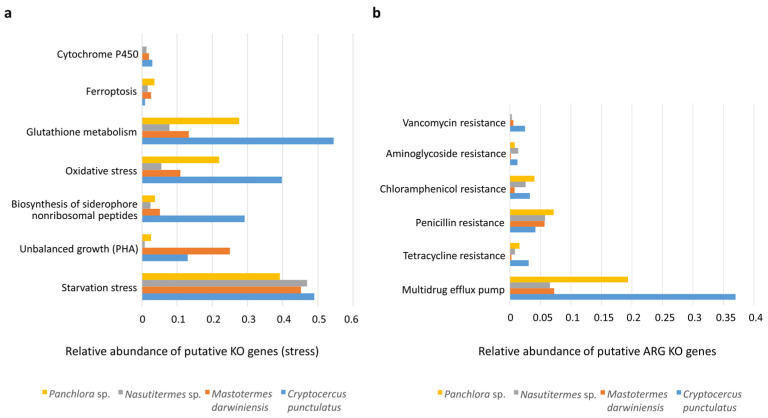
Putative KO genes related to stress and antibiotic resistance. (**a**) Bar graph depicting the relative abundance of putative KO genes involved in various cellular stress response mechanisms across the gut microbiomes of *Panchlora* sp. (yellow), *Nasutitermes* sp. (grey), *M. darwiniensis* (orange), and *C. punctulatus* (blue). The relative abundance of these KO genes could reflect the adaptive capacity of microbial communities to cope with environmental stressors and maintain cellular homeostasis within the insect gut environment. (**b**) Bar graph showing the relative abundance of putative ARG (antibiotic resistant genes) KO genes conferring resistance to different classes of antibiotics in the same four insect species. Resistance mechanisms evaluated include genes for vancomycin, aminoglycosides, chloramphenicol, penicillin, and tetracycline, as well as genes encoding multidrug efflux pumps (panel (**b**)).

The insect gut is a major site of detoxification and stress response. Cytochrome P450 monooxygenases (P450s) catalyze key redox reactions and detoxify a wide range of xenobiotics [26]. P450 genes were detected in all insects examined, indicating potential for xenobiotic degradation; however, they represented less than 1% of total KO annotations, suggesting that detoxification plays a relatively minor role compared with pathways such as carbohydrate metabolism. Other oxidative stress–related enzymes—including superoxide dismutases, catalases, peroxidases, and components of the glutathione system—were present in all insects, with particularly high representation in cockroaches, especially *C. punctulatus* (Figure 5a) [27,28]. Ferroptosis, an iron-dependent, lipid peroxidation–associated form of regulated cell death [29], was slightly higher in *Panchlora* sp.

Despite access to dietary carbon sources, nutrient starvation stress markers (e.g., K03087, K03088) were detected in both cockroaches and termites. In addition, genes involved in the synthesis of the reserve polymer polyhydroxyalkanoate (PHA) (e.g., K03821, K00023, K05973) were observed in *M. darwiniensis* and *C. punctulatus*, indicative of unbalanced growth (Figure 5a). PHA accumulation is typically stimulated under nutrient limitation (e.g., P, N, Fe) combined with excess carbon availability. The co-occurrence of starvation response and PHA synthesis pathways suggests that gut microbiota experience intermittent nutrient deprivation, likely due to fluctuations in dietary intake or competition within the densely populated gut environment.

Antibiotic resistance KO genes associated with vancomycin, aminoglycoside, chloramphenicol, penicillin, tetracycline, and multidrug efflux pumps were detected in all insects examined (see Figure 5b). Among these, multidrug resistance pumps and penicillin resistance genes were most abundant. Cockroaches exhibited a greater number of antibiotic resistance-related KO genes than termites (Figure 5b).

## 4. Discussion

### 4.1. Bacterial Gut Patterns from Cryptocercus punctulatus

The symbiotic gut microbiota is integral to insect physiology, particularly in wood-feeding termites and the xylophagous cockroach *Cryptocercus*, which rely on microbial partners to process nutrient-poor, lignocellulosic diets. These gut microorganisms facilitate the digestion of recalcitrant substrates, synthesize essential vitamins and nutrients, and provide additional functions fundamental to host fitness [4,30,31,32].

The methodologies employed—amplicon sequencing targeting the V1–V2 and V3–V4 regions of the 16S rRNA gene and whole metagenome shotgun sequencing—are known to introduce inherent biases that can influence the detection and quantification of microbial taxa. Amplicon sequencing depends on PCR primer specificity, which may preferentially amplify certain taxa while underrepresenting or missing others, particularly those with mismatches in primer-binding regions. Conversely, shotgun metagenomics sequences all DNA fragments present in a sample without primer bias, but its taxonomic resolution and sensitivity can be affected by sequencing depth, host DNA contamination, and bioinformatic limitations [33,34]. The V1–V2 region generally exhibits higher species-level taxonomic richness and diversity than the V4–V5 region. Certain bacterial genera, such as some Bacillota and Actinobacteriota, are more abundantly detected with V1–V2 primers, whereas V4–V5 may underrepresent these taxa or show different relative abundances [35]. Across the *C. punctulatus* samples analyzed in this study, the relative abundance of dominant bacterial taxa Bacteroidota, Bacillota, and Pseudomonadota varied among samples. Despite methodological biases, Pattern-1 encompassed samples from both the V1–V2 and V4–V5 regions (Cp, Cp_1, Cp_2, and Cp_3) (Figure 1). However, no statistically significant differences at the phylum level were detected between pattern-1 and pattern-2 or between Pattern-1 and Pattern-3 (Kolmogorov–Smirnov test, *p* > 0.05) (Figure 1). This suggests a degree of compositional flexibility within the gut community, with multiple stable bacterial configurations persisting without compromising functional integrity.

Beyond methodological effects, another factor potentially influencing the observed profiles the type of wood from which specimens were collected. The forests of North Carolina and Virginia, where *C. punctulatus* specimens were collected (Mount Collins, South Mountains, Haywood County, and Mountain Lake), belong to the Southern and Central Appalachian mixed hardwood ecoregion, characterized by similar dominant tree taxa. Although tree species differ slightly among sites, the dominant vegetation across these forests consists primarily of hardwood taxa (oak, maple, birch, hickory, and beech) with conifers such as fir and spruce present only at higher elevations. These tree species share broadly similar lignocellulosic compositions, characterized by high cellulose and hemicellulose content and secondary compounds typical of temperate hardwoods. Therefore, substrate quality and nutrient availability for *C. punctulatus* may be comparable across sites [36], suggesting that diet may not be the primary driver of gut bacterial community structure.

*Cryptocercus punctulatus* is currently divided into four known karyotype groups (2*n* = 37, 39, 43 and 45). Despite genetic differences among these groups, no ecological or habitat use differences have been reported within their examined range [37]. The specimens used in this study were Cp_1 (2*n* = 43), Cp_2 (2*n* = 43), and Cp_3 (2*n* = 45) [20]. Although they had different karyotypes, they exhibited similar bacterial community patterns, supporting the conclusion that neither host genetic background nor local diet strongly influences phylum-level microbiota structure in these populations. Instead, ecological and functional stability—likely reflecting evolutionary pressures for efficient lignocellulose degradation appears to conserve core gut community features across host genetic variants and localities.

Such flexibility is likely rooted in the early establishment of the microbiota during *Cryptocercus* development. In these subsocial cockroaches, proctodeal trophallaxis from adults to juveniles ensures vertical transmission of symbionts and the formation of core microbial communities [37]. The complete symbiotic microbiota assembles sequentially and is not finalized until the third instar, when individuals attain nutritional independence, although they continue to maintain contact with adults [38].

The observed interindividual variation likely reflects the inherent ecological plasticity of the gut microbiome. These findings reinforce the view that the *C. punctulatus* gut microbiota achieves a balance between stability and compositional flexibility, fitting within a host–microbe ecological framework where multiple taxonomic assemblages can sustain similar functional outcomes. This resilience likely represents an adaptive feature of xylophagous symbioses, ensuring consistent host nutrition despite environmental fluctuations or experimental variation.

Samples maintained under laboratory conditions (Cp_5, Cp_shotgun) did not differ significantly from those collected in nature, suggesting that laboratory maintenance does not substantially disrupt the *Cryptocercus* gut community. Previous studies have reported that insects kept in laboratory environments over extended periods often develop gut communities differing in both diversity and composition from those of wild-caught individuals, largely due to dietary and environmental changes [39,40]. This observation is particularly relevant for comparative analyses of Cp_shotgun samples for taxonomic and functional features relative to metagenomic datasets from wild-caught insect species, consistent with previous findings on cockroach gut microbiomes [41].

### 4.2. Shotgun Taxa Metagenome of Cryptocercus punctulatus and Other Xylophagous Dyctioptera

The Cp_shotgun metagenome from *C. punctulatus* was compared with metagenomic datasets from other xylophagous Dictyoptera. The specialized bacterial genera observed in each insect appear to be characteristic features enabling them to thrive on lignocellulosic diets [42,43]. At the genus level, the *C. punctulatus* gut is dominated by *Raoultella*, *Klebsiella*, *Pseudomonas*, *Azovibrio* and *Dysgonomonas*. These genera are functionally relevant to lignocellulose-based diets, as they are implicated in complex polysaccharide degradation, short-chain fatty acid production, vitamin biosynthesis, and nitrogen fixation. Such capabilities are consistent with the nutritional demands of a wood-feeding lifestyle and align with previous reports linking these taxa to cellulose and hemicellulose breakdown in other arthropod hosts [44,45,46].

Principal coordinate analysis at the family-level revealed that *C. punctulatus* harbors a distinct bacterial assemblage relative to both termites and the distantly related cockroach *Panchlora* sp. This divergence likely reflects host-specific gut environments, including differences in redox gradients, metabolic niches and nutrient availability, together with the unique evolutionary history of symbiosis in each lineage. Distinct microbial community organizations were observed among the insects studied. “Lower” termites, such as *M. darwiniensis*, maintain a mixed protist–bacteria symbiosis, while higher termites (e.g., *Nasutitermes* sp). exhibit bacteria-dominated communities enriched in taxa specializing in hemicellulose and chitin degradation. *C. punctulatus* preserves a protist–bacteria consortium similar to that of lower termites, but with distinctive bacterial contributions reflecting its cockroach ancestry. Wood digestion in Dictyoptera may, therefore, have arisen through both shared symbiotic legacies and independent innovations in host–microbe associations [3,15].

### 4.3. Functional Nutritional Shotgun Metagenome of Cryptocercus punctulatus and Other Xylophagous Dyctioptera

Across all xylophagous Dictyoptera, gut microbiomes encode extensive repertoires of carbohydrate-active enzymes (CAZymes) for cellulose, hemicellulose, pectin, and starch hydrolysis, whereas canonical lignin-degrading enzymes (laccases, manganese peroxidases, and lignin peroxidases) were essentially absent, with only low-abundance *kat*G and *lig*D homologs detected. This supports the notion that enhancing polysaccharide accessibility rather than lignin mineralization suffices for wood digestion [47]. *Nasutitermes* sp. displayed the highest xylanolytic and chitinolytic potential, predominantly mediated by Spirochaetota and Fibrobacteriota CAZymes [48], likely reflecting fungal biomass degradation within decaying wood [49,50]. In nature, the main chitin degraders are bacteria from the phyla Bacteroidota and Bacillota, which produce CAZymes capable of lysing fungal cell walls and insect cuticles, composed of protein and chitin [50,51]. Specifically, these bacteria putatively generate mycolytic enzymes that break down chitin in these structures [50] (Figure 4a).

Inositol phosphate metabolism encompasses biochemical pathways involved in the synthesis, breakdown, and signaling of inositol phosphates, which play critical roles in cell signaling, membrane trafficking, and gene regulation [52]. Inositol phosphate metabolic pathways were less represented in cockroaches compared with termites (Figure 4a). Similar results were observed for glyoxylate and dicarboxylate metabolism, which enable organisms to utilize acetate as an energy source when glucose availability is limited. The glyoxylate cycle converts acetyl-CoA to malate while bypassing carbon dioxide loss, allowing greater carbon incorporation into biomass. Oxaloacetate and malate provide carbon skeletons for amino acid synthesis. Thus, the glyoxylate cycle supplies simple sugars, biosynthetic precursors, and amino acid substrates when glucose is scarce [53]. The reduced representation of these pathways in cockroaches compared with termites suggests differences in acetate and glucose metabolism between these groups, implying that cockroaches may depend more heavily on glucose metabolism.

Insects have evolved various strategies to cope with low dietary nitrogen, including symbiotic nitrogen fixation, efficient nitrogen recycling, and optimization of nitrogen intake. Key nitrogen-fixing symbionts include diazotrophic bacteria such as Spirochaeota and Bacteroidota, which provide fixed nitrogen to their hosts [13]. Insects also recycle nitrogenous wastes such as uric acid, urea, and ammonia via glutamine synthetase and glutamate synthase pathways. They excrete nitrogenous waste in forms such as uric acid, ammonia, allantoin and allantoate via the Malpighian tubules and hindgut. Other nitrogen acquisition mechanisms include coprophagy (fecal consumption), proctodeal trophallaxis (anus-to-mouth feeding), predation or cannibalism, and ingestion of shed cuticles [25,51]. Termites relied chiefly on diazotrophic bacteria for nitrogen fixation, whereas *Cryptocercus* exhibited elevated gene counts for uric-acid and urea recycling and non-protein amino acid (e.g., taurine) metabolism, indicating a shift toward waste-derived nitrogen salvage [54,55,56] (Figure 4b).

### 4.4. Functional Stress and Antibiotic Shotgun Metagenome of Cryptocercus punctulatus and Other Xylophagous Dyctioptera

Changes in the gut microbial communities of xylophagous insects can occur in response to environmental stressors. Factors such as diet, temperature extremes, or exposure to insecticides can alter the diversity and composition of gut bacteria [57,58,59]. The microbiota must also tolerate intrinsic gut conditions, including toxic plant compounds (e.g., alkaloids, terpenoids, phenols, etc.), oxygen exposure, variable pH, nutrient limitation, and competitive exclusion conditions that impose substantial stress on microorganisms.

Herbivorous insects have evolved strategies to detoxify compounds encountered in their habitats, including enzymes such as carboxylesterase, glutathione-S-transferases (GSTs), and cytochrome P450 monooxygenases (CYP450s Cytochrome P450 enzymes, a large family of heme-containing oxidases, occur across bacteria, fungi, plants, and animals. After ingestion, plant tissues enter the insect digestive tract, where the gut bacterial community aids in digestion, nutrient absorption, and detoxification of harmful phytochemicals [60]. For example, *Pseudomonas* species can degrade monoterpenes and diterpene acids [61]. Cytochrome P450 KO genes had lower relative abundance in the metagenomes of both cockroaches and termites, suggesting that the microbiota did not play a fundamental role in detoxification, which may instead be mediated by the insects’ own enzymes—a pattern observed in several insect taxa [28,62].

Oxygen-rich tissues surround insect guts, as air is delivered through the tracheal system. Oxygen permeates into the hindgut contents up to 150–200 mm below the epithelium, where the gut microbiota depletes through respiration, generating a microoxic periphery surrounding an anoxic center [62,63]. A stable, physiological state in the host may support high microbial diversity, likely maintaining a sharp O_2_–H_2_ gradient within the gut [63,64]. Enzymes that combat oxidative stress such as superoxide dismutases, catalases, peroxidases, and glutathione, as well as the oxidative process of ferroptosis, were detected across all insects examined. Cockroaches, particularly *C. punctulatus*, exhibited notably high levels of oxidative stress-related enzymes, with catalase genes showing greater relative abundance than those for superoxide dismutase (SOD) (Figure 5a). A similar pattern has been reported in the American cockroach *Periplaneta americana* [28]. The abundance of these redox-regulating components in cockroaches suggests adaptability to shifts in gut redox states, which may fluctuate depending on lignocellulose consumption [63,65].

Antibiotic resistance genes (ARGs) are widespread in diverse environments—soil, freshwater, marine systems, wastewater treatment plants, and even pristine habitats—reflecting their ancient evolutionary origins [66,67]. However, the extensive use of antibiotics has greatly increased ARG prevalence. In this context, wildlife can serve as indicators of environmental pollution by antibiotic resistance genes, especially in densely populated human areas [68,69,70].

Genes conferring resistance to vancomycin, aminoglycosides, chloramphenicol, β-lactams, tetracyclines, and multidrug efflux pumps were detected across all samples, with cockroach metagenomes harboring the greatest resistome diversity (Figure 5b). Because the analyzed insects originated from pristine environments with minimal anthropogenic influence, their resistomes were likely shaped mainly by intrinsic microbial interactions and natural selective pressures rather than direct human activity. These results provide a valuable baseline for assessing naturally occurring ARG levels in environmental microbial communities.

Termites analyzed in this study exhibited lower relative ARG abundance than cockroaches, likely due to ecological and biological differences, including their eusocial lifestyle. The specialized feeding behaviors of termites actively maintain microbiome stability and limit ARG acquisition [71]. Cockroaches, in contrast, exhibit more opportunistic ecological strategies, exposing them to broader environmental microbial reservoirs. While *Cryptocercus* cockroaches also engage in proctodeal feeding, their social structures are less rigid, and their environmental interactions are more variable. This lifestyle promotes a dynamic gut microbiome capable of incorporating environmental bacteria, including those carrying ARGs. The detection of ARGs in *C. punctulatus* and *Panchlora* sp. may indicate environmental dissemination from distant human sources, though their role in ARG transmission is likely limited compared to urban insects. Urban environments—with high antibiotic use and contamination—promote ARG proliferation, as seen in synanthropic cockroaches such as *Blattella germanica* [72]. Studies in gregarious cockroaches, such as *Pycnoscelus surinamensis*, have shown increased ARG abundance following antibiotic exposure, with resistance subsequently transmitted to untreated populations through social interaction [73].

The gut microbiota of cockroaches—from both forest and urban environments—thus represents a potential sentinel for anthropogenic antibiotic pollution and mediator of gene flow between ecological reservoirs [74].

## 5. Conclusions

This study provides valuable insights into the gut microbiota of the wood-feeding cockroach *C. punctulatus* and its functional comparison with other xylophagous Dictyoptera. Several limitations should be considered. Methodological differences, such as the use of amplicon versus shotgun metagenomics, may introduce biases in microbial detection and quantification, potentially affecting the precision of taxonomic and functional comparisons. Additionally, the study focused primarily on bacterial communities, as protist detection had low resolution in metagenomic analyses, leaving some symbiotic roles unexplored. Future research should aim to refine metagenomic methods to improve eukaryotic microbiota resolution and expand comparative analyses across broader ecological settings. Despite contrasting community compositions, xylophagous cockroaches and termites exhibit remarkable functional convergence in carbon and nitrogen metabolism, while cockroaches appear to serve as hotspots for environmentally acquired antibiotic resistance genes. The high antibiotic resistance observed in cockroaches compared with termites suggests environmental acquisition, highlighting the need for comparative studies between urban and pristine habitats to better understand resistance gene sources and dissemination dynamics. Understanding the composition and functional capabilities of xylophagous insect gut microbiomes not only advances our knowledge of host–microbe coevolution, but also carries significant implications for sustainable agriculture—informing biotechnological strategies for lignocellulose bioconversion and nutrient cycling—and for public health, given that the spread of antibiotic resistance genes in environmental reservoirs may influence the emergence of resistant pathogens in agricultural and urban ecosystems.

## Figures and Tables

**Table 1:** *C. punctulatus* normobiota amplicon sequencing samples.

Sample	Field Collected	16S rRNA	Intestinal Tract	Valid Reads	Silva Clustering (OTU)
Cp	North Carolina (NC) or Virginia (VA), USA	V3–V4	Entire gut	148,434	40,364
Cp_1	NC (Mt. Collins), USA	V1–V2	Hindguts	12,352	10,513
Cp_2	VA (Mountain Lake), USA	V1–V2	Hindguts	10,297	9034
Cp_3	NC (South Mountains) USA	V1–V2	Hindguts	9322	4445
Cp_4	NC (Heywood Country) USA	V3–V4	Hindguts	36,158	10,388
Cp_5	VA (USA) and maintained in laboratory conditions	V1–V2	Entire gut	5221	2356

**Table 2:** Shotgun metagenomes of different xylophagous Dictyoptera species.

Dictyoptera	Collected	JGI Database Gold	Annotated Gens	% Assembled (Genes KO)
*Mastotermes darwiniensis*	Townsville, Australia	Ga0068304	1,421,148	6.80
*Nasutitermes* sp.	Murphy’s Creek, Queensland, Australia	Ga0072940	1,121,956	18.49
*Panchlora* sp.	Gamboa Forest, Panama	Ga0026008	108,739	33.91

## Data Availability

Data availability in the JGI database Gold pj. Ga0134290 (*C. punctulatus*) (https://gold.jgi.doe.gov/analysis_project?id=Ga0134290 (accessed on 1 November 2025).

## Supplementary Materials

The following supporting information can be downloaded at: https://www.mdpi.com/article/10.3390/insects16111128/s1, Figure S1: Principal coordinates analysis of the community distribution of several *C. punctulatus* samples based on phyla relative abundance obtained by amplicon sequencing made by statgraphics centurion v18.0 program. Cp (V3–V4 region); Cp_1–Cp_3 (V1-V2 region); Cp_4 (V3-V4 region); and Cp_5 (V1-V2 region) (see Table 1). Figure S2: Principal coordinates analysis of the community distribution. (a) Based on taxonomy annotation of shotgun metagenomes processed in the JGI database. (b) Based on functional annotation of shotgun metagenomes processed in the JGI database (panel b) [http://www.jgi.doe.gov/].
