# Supplementary material for: Disentangling Gut Bacterial Community Patterns in Cryptocercus punctulatus and Comparing Their Metagenomes with Other Xylophagous Dyctioptera Insects
Source: Insects. 2025 Nov 4;16(11):1128. doi: 10.3390/insects16111128 (PMC12653833; doi:10.3390/insects16111128)
Supplement: Supplementary file 1 [file insects-16-01128-s001.zip › insects-3929748-supplementary.pdf]

Supplemental material

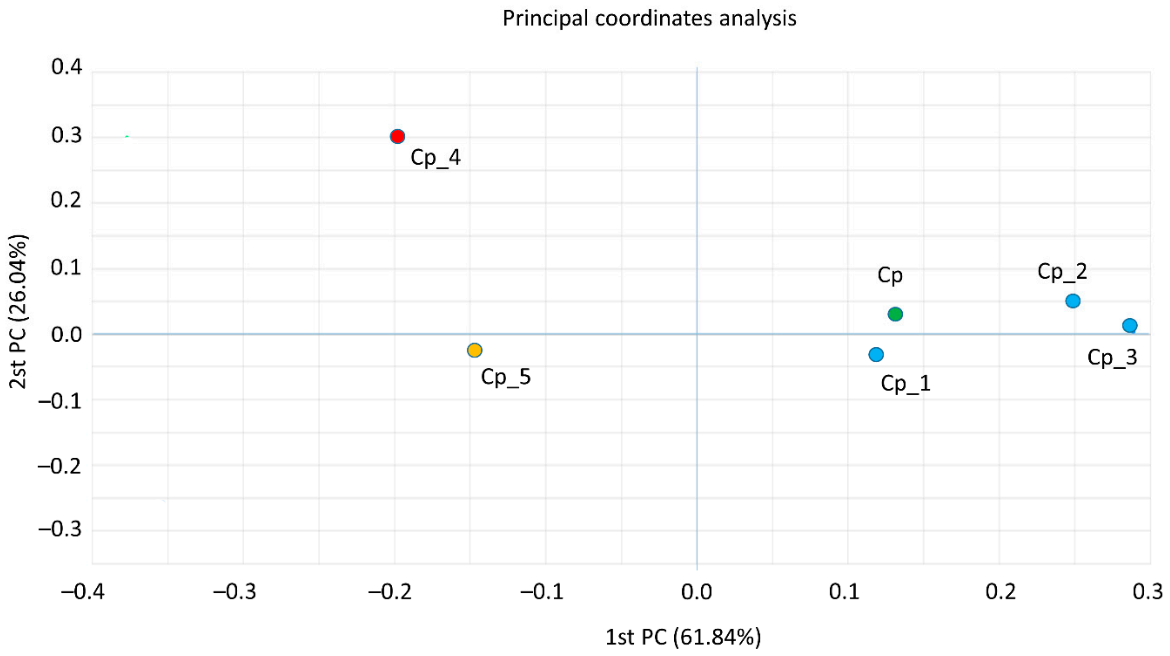

Supplementary Figure S1. Principal coordinates analysis of the community distribution of several *C. punctulatus* samples based on phyla relative abundance obtained by amplicon sequencing made by statgraphics centurion v18.0 program. Cp (V3–V4 region); Cp_1–Cp_3 (V1-V2 region); Cp_4 (V3-V4 region); and Cp_5 (V1-V2 region) (see Table 1).

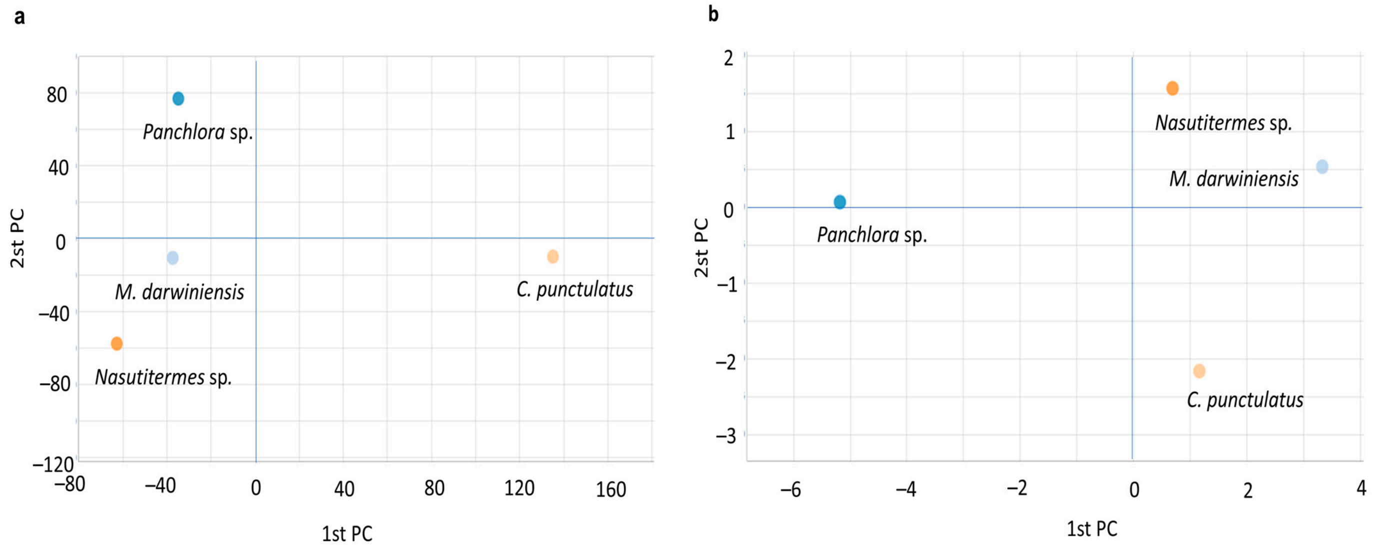

Supplementary Figure S2 Principal coordinates analysis of the community distribution. (a) Based on taxonomy annotation of shotgun metagenomes processed in the JGI database. (b) Based on functional annotation of shotgun metagenomes processed in the JGI database (panel b) [http://www.jgi.doe.gov/].
